# Antiretroviral medications disrupt microglial phagocytosis of β-amyloid and increase its production by neurons: Implications for HIV-associated neurocognitive disorders

**DOI:** 10.1186/1756-6606-4-23

**Published:** 2011-06-07

**Authors:** Brian Giunta, Jared Ehrhart, Demian F Obregon, Lucy Lam, Lisa Le, JingJi Jin, Francisco Fernandez, Jun Tan, R Douglas Shytle

**Affiliations:** 1 Neuroimmunology Laboratory, Department of Psychiatry and Neurosciences, College of Medicine, University of South Florida, Tampa, FL, 33612, USA; 2 Department of Molecular Medicine, Department of Psychiatry and Neurosciences, College of Medicine, University of South Florida, Tampa, FL, 33612, USA; 3 Silver Child Development Center, Department of Psychiatry and Neurosciences, College of Medicine, University of South Florida, Tampa, FL, 33612, USA; 4 Center Excellence in Aging and Brain Repair, Department of Neurosurgery, College of Medicine, University of South Florida, Tampa, FL, 33612, USA; 5 Department of Pharmacology and Physiology, College of Medicine, University of South Florida, Tampa, FL 33612, USA

**Keywords:** antiretrovirals, microglial cells, HIV, cognitive disorders

## Abstract

Up to 50% of long-term HIV infected patients, including those with systemically well-controlled infection, commonly experience memory problems and slowness, difficulties in concentration, planning, and multitasking. Deposition of Aβ plaques is also a common pathological feature of HIV infection. However, it is not clear whether this accumulation is due to AD-like processes, HIV-associated immunosuppression, Tat protein-induced Aβ elevations, and/or the effects of single highly active antiretroviral therapy (ART). Here we evaluated the effects of several ART medications (Zidovudine, Lamivudine, Indinavir, and Abacavir) alone and in combination on: *1) *Aβ_1-40, 42 _generation in murine N2a cells transfected with the human "Swedish" mutant form of APP; *2) *microglial phagocytosis of FITC-Aβ_1-42 _peptides in cultured murine N9 microglia. We report for the first time that these antiretroviral compounds (10 μM) generally increase Aβ generation (~50-200%) in SweAPP N2a cells and markedly inhibit microglial phagocytosis of FITC-Aβ_1-42 _peptides in murine microglia. The most significant amyloidogenic effects were observed with combined ART (p < 0.05); suggesting certain ART medications may have additive amyloidogenic effects when combined. As these antiretroviral compounds are capable of penetrating the blood brain barrier and reaching the concentrations employed in the *in vitro *studies, these findings raise the possibility that ART may play a casual role in the elevated Aβ found in the brains of those infected with HIV. Therefore these compounds may consequently contribute to cognitive decline observed in HIV associated neurocognitive disorders (HAND).

## Introduction

Cognitive impairment occurs in a substantial (15-50%) proportion of HIV-infected patients [1-3]. HIV-associated dementia (HAD) represents the most severe form [4]. With the introduction of antiretroviral therapy (ART), the incidence of HAD has dramatically decreased. In the past several years, patients--both long-term infected and treated--including those with systemically well-controlled infection, began to report milder memory problems and slowness, difficulties in concentration, planning, and multitasking; collectively termed HIV-associated neurocognitive disorders (HAND; [2]).

Although the pathological mechanism underlying HAND is unclear, an abundance of clinical and laboratory investigations suggest that HIV proteins, advanced age, and co-morbid neurodegenerative disease may interact in an additive or even synergistic manner resulting in the clinical presentation of this disorder [5,6]. This is concerning as there is an estimated 60,000 HIV-infected individuals over the age of 50 and 10,000 over the age of 65. Furthermore, it has been predicted that 50% of prevalent acquired immunodeficiency syndrome (AIDS) cases in the United States will fall into this older age group by the year 2015 [7].

Interestingly, with such increased survival times imparted by ART, the prevalence of HAND is on the rise. At present, it is not clear whether this increased prevalence is due to the intrinsic risk of developing dementia with age or due to other direct or indirect factors of ART. In a recent clinical study, it was found that neurocognitive functioning significantly improved after immune competent, HIV-infected patients discontinued ART treatment. Moreover, this improvement continued in the patients remaining off ART over the nearly two year period of follow-up. Additionally, there was a lack of substantial neurocognitive improvement with resumption of ART [8].

There are several potential explanations underlying this clinically meaningful finding. One of these is that ART may lead to neurotoxicity [9] which is manifested in part by dysregulated neuronal amyloid precursor protein (APP) processing and concurrent deposition of amyloid beta (Aβ) plaques in the brain.

While extracellular amyloid plaques are the primary amyloid pathology in Alzheimer's disease (AD), intraneuronal amyloid accumulation or perivascular diffuse amyloid depositions are more of a feature of HAND [3]. Strong evidence indicates this increased amyloid deposition in the brain of HIV-1-infected patients [10] as opposed to true full blown AD-like pathology. A correlation between the years of infection and amyloid deposition has also been shown [11]. Further, this amyloid deposition is most prevalent in the hippocampus and frontal lobe regions [12], and also observed in pyramidal neurons and along axonal tracks in HIV infected patients. Importantly, patients with HIV-associated encephalitis (HIVE) had higher levels of intraneuronal Aβ immunoreactivity compared to HIV-1 patients without HIVE. In addition, intracellular deposition of Aβ correlated with age in the group of patients with HIVE [13]. Finally, HAND in older populations is at least partially linked to early signs of β-amyloidosis observed in AD, further demonstrating the importance of Aβ deposition for the clinical outcome of HIV-1 infection. These studies raise questions regarding possible iatrogenic mechanisms involved in the pathogenesis of both HAND and/or AD as a co-morbid neurodegenerative [3,13].

In the present pilot study, we investigate the potential effects of various ART medications on neuronal Aβ production and clearance by microglial phagocytosis in murine N2a cells transfected with the human "Swedish" mutant form of APP (SweAPP N2a cells) and N9 microglia cultures, respectively.

## Methods

### Antiretrovirals

The following reagents were obtained through the NIH AIDS Research and Reference Reagent Program, Division of AIDS, NIAID, NIH: Zidovudine (AZT; Cat.# 3485), Abacavir (Cat.# 4680), Indinavir (Cat.# 8145), and Lamivudine (3TC; Cat# 8146).

### Neuronal Aβ Production Assay

SweAPP N2a cells were seeded at 1 × 10^5 ^cells/well (n = 2 for each condition) in 24-well tissue-culture plates containing 0.5 mL of complete medium (MEM medium supplemented with 10% fetal calf serum). Cells were differentiated prior to treatment with cAMP in 0.5 mL Neurobasal media for 4 hours. Following differentiation, these cells were untreated (control vehicle; PBS) or treated with ART medications both alone (10 μM) and in combination (10 μM) for 18 hours. Aβ_1-40, 42 _peptides were detected directly from the conditioned media and quantified in these samples using Aβ_1-40, 42 _ELISA kits in accordance with the manufacturer's instructions.

### Microglial Phagocytosis Assay

N9 microglia were seeded at 1 × 10^5 ^cells/well (n = 4 for each condition) in 24-well tissue-culture plates containing 0.5 mL of complete medium (RPMI 1640 medium supplemented with 5% fetal calf serum). In the presence "aged" Aβ_1-42 _peptide conjugated with FITC (supplied by BioSource, used at 300 nM dissolved in dH_2_0 and pre-incubated for 24 h at 37°C) microglia were co-treated with retroviral drugs both alone (10 μM) and in combination (10 μM) for 0, 30, 60, 120, and 180 minutes. For fluorometric analysis, in parallel treatments, the microglia were rinsed 3 times with complete medium after collection of cell supernatants and subjected to cell lysate preparation [14,15]. The total cellular protein of all groups was quantified and adjusted using the Bio-Rad protein assay. Extracellular and cell associated FITC-tagged Aβ was quantified using an SPECTRAmax GEMINI microplate fluorometer (Molecular Devices Corp.) with an emission wavelength of 538 nm and an excitation wavelength of 485 nm. In addition, in parallel 24-well tissue-culture plates, microglial cells were incubated at 4°C with FITC-conjugated Aβ with or without the various antiretroviral treatments as controls for non-specifically incorporated Aβ. Microglial cells were rinsed 3 times in Aβ-free complete medium, and the media was exchanged with fresh Aβ-free complete medium for 10 min both to allow for removal of non-incorporated Aβ and to promote concentration of the Aβ into phagosomes. The relative mean fluorescence values for each sample at 37°C and 4°C at the indicated time points were determined by fluorometric analysis. Relative mean values were calculated as: (mean fluorescence value for each sample at 37°C - mean fluorescence value for each sample at 4°C). In this manner, both extracellular and cell associated FITC-labeled Aβ were quantified.

### Statistical Analysis

All data were normally distributed; therefore, in instances of single mean comparisons, Levene's test for equality of variances followed by *t*-test for independent samples was used to assess significance. In instances of multiple mean comparisons, analysis of variance (ANOVA) was used, followed by *post-hoc *comparison using Bonferonni's method/correction. Alpha levels were set at 0.05 for all analyses. The statistical package for the social sciences release 10.0.5 (SPSS Inc., Chicago, IL, USA) was used for all data analysis.

## Results

### ART medications increase Aβ generation in cultured SweAPP N2a cells

**Figure 1:**
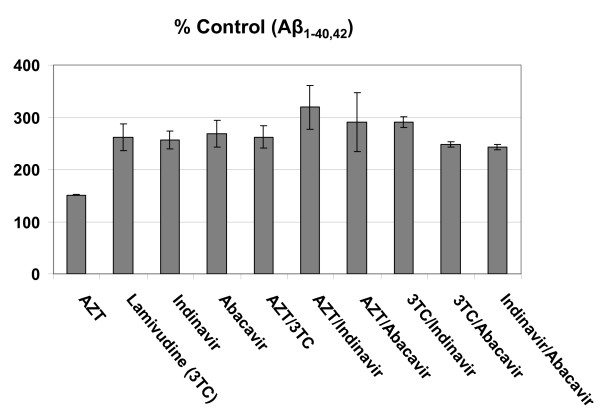
**ART medications increase Aβ generation in cultured neuronal cells**. Total Aβ _1-40,42 _peptides were analyzed in conditioned media from SweAPP N2a cells by ELISA (*n *= 2 for each condition). Data are represented as a mean ± SEM percentage of Aβ _1-40,42 _peptides secreted 18 hours after antiretroviral treatment relative to control (untreated). One-way ANOVA followed by *post hoc *comparison revealed significant increases in Aβ _1-40,42 _production following 3TC/Indinavir, 3TC/Abacavir, and Indinavir/Abacavir treatements (concentration of 10 μM for each individual compound; p < 0.05).

ART regimens are typically comprised of two major drug classes: protease inhibitors (PIs) and inhibitors of reverse transcriptase. The latter is subdivided into nucleoside reverse transcriptase inhibitors (NRTIs) and non-nucleoside reverse transcriptase inhibitors (nNRTIs). To examine the potential effects of a typical ART regimen on amyloidosis, we treated SweAPP N2a cells with 10 μM concentrations of various antiretroviral compounds both alone and in combination for 18 hours. Following ELISA of the cultured media, we found that all compounds generally increased (~50-200%) Aβ_1-40, 42 _production (Figure 1). Interestingly, significant increases were observed when antiretroviral compounds were used in combination (*p < 0.05*, Figure 1). Although we observe considerable increases in Aβ_1-40, 42 _generation with all of the antiretroviral compounds, it appears that combinations of the NRTIs Lamivudine (3TC) and/or Abacavir, with, the PI Indinavir, exert the most significant amyloidogenic effects. Notably, the NRTI Zidovudine (AZT) conferred significantly less Aβ _1-40,42 _production as compared to the antiretroviral combinations mentioned above (*p < 0.05*; Figure 1). Taken together, the data suggests that iatrogenic mechanisms may in fact contribute to HAND and AD-like pathology in HIV-infected individuals.

### ART medications inhibit microglial phagocytosis of Aβ _1-42 _peptides

**Figure 2:**
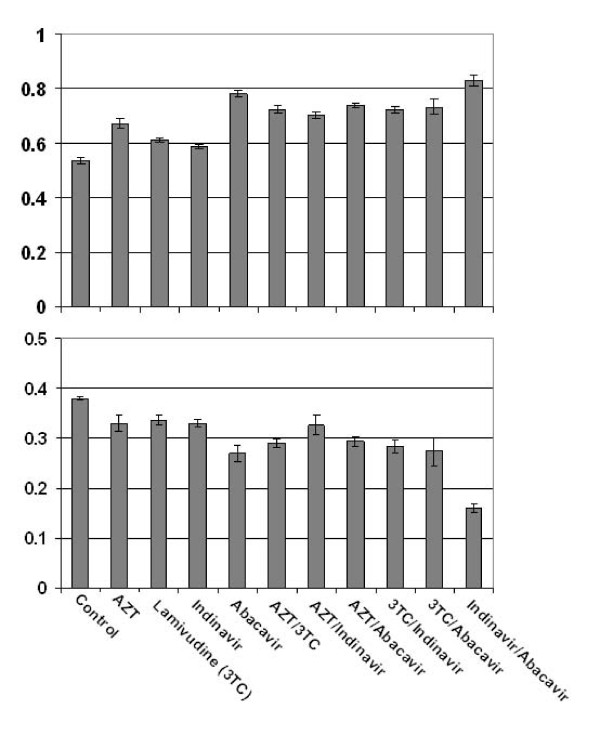
**ART medications inhibit microglial phagocytosis of Aβ_1-42 _peptides**. N9 microglia were treated with "aged" FITC-tagged Aβ _1-42 _(300 nM) in complete medium for 120 min in the presence of 10 μM concentrations of antiretrovirals. Total FITC-Aβ_1-42 _peptides were analyzed by fluorescence from conditioned media (extracellular; top) and cell lysates (phagosomal/cell associated; bottom). Data are represented as the relative mean ± SEM fluorescence (n = 4 for each condition presented). When measuring extracellular FITC-tagged Aβ _1-42_, one-way ANOVA followed by *post hoc *comparison showed significantly higher levels following all antiretroviral treatments, as compared to control (p < 0.05). When measuring cell associated FITC-tagged Aβ _1-42_, one-way ANOVA followed by *post hoc *comparison showed significantly lower levels following Indinavir, Abacavir, AZT/3TC, AZT/Abacavir, 3TC/Indinavir, and Indinavir/Abacavir, as compared to control (p < 0.05).

To determine whether ART could affect microglial clearance of Aβ and further promote amyloidosis, we performed a phagocytosis assay with N9 cells in the presence of antiretroviral compounds both alone and in combination. Following detection of FITC-tagged Aβ_1-42 _in extracellular and cell associated fractions, we again found that all compounds generally inhibited microglial phagocytosis/clearance (Figure 2). All antiretroviral compounds significantly inhibited microglial phagocytosis of Aβ_1-42 _peptides as determined by high levels of peptide remaining in the cultured media (extracellular) (*p < 0.05*, Figure 2a). In addition, a majority of the compounds tested also significantly reduced levels of phagosomal (cell associated) Aβ_1-42 _(*p < 0.05*, Figure 2b). Also, when comparing cell associated Aβ_1-42 _levels of the Indinavir/Abacavir combination to levels of these compound alone, the differences suggest the combination of this PI and NRTI is additive in nature (*p < 0.05*; Figure 2b). Importantly, when comparing the levels of extracellular Aβ_1-42 _(Figure 2a) to that of cell associated (Figure 2b) we can see that the phagocytosis/clearance profiles are relatively congruent for each treatment condition. That is to say, when a given treatment maintains high levels of extracellular Aβ_1-42_, the corresponding cell associated levels are relatively low. Not only does this apparent relationship between extracellular and cell associated Aβ_1-42 _levels confirm the accuracy of the assay, but also furthers the overall significance of the inhibition of microglial phagocytosis by the antiretrovirals.

## Discussion

Therapy with at least three ART medications has been standard treatment for HIV infected patients for since approximately 1995 [16]. Indeed combination ART has dramatically reduced medical morbidity and mortality with HIV infection, but high rates of HAND continue to be reported [17]. Heaton and colleagues sought to determine neurocognitive impairment in large groups of HIV + and HIV - participants from the pre-combination ART era (1988-1995; N = 857) and combination ART era (2000-2007; N = 937). The rates of impairment increased with successive disease stages in both eras: 25%, 42%, and 52% in pre-ART era and 36%, 40%, and 45% in combination ART era. In the medically asymptomatic stage, neurocognitive impairment was significantly more common in the ART era; indicating a possible iatrogenic mechanism whereby ART is salutary in the periphery, but possibly in some way deleterious in the CNS [17]. Furthermore, the pattern of neurocognitive impairment also differed. Patients from the pre-ART era had more impairment in motor skills, cognitive speed, and verbal fluency. On the other hand, ART era patients suffered more memory (learning) and executive function deficits. Importantly, this study showed high rates of mild neurocognitive impairment persist at all stages of HIV infection, despite adequate viral suppression and immune reconstitution with combination ART [17].

In this context, we sought to identify the risk of adverse neuropathological side effects from various ART regimens *in vitro*. Here, we elucidate a potential mechanism whereby antiretroviral compounds may have neurotoxic effects, both alone and in combination. This may contribute to the neurological complications that are associated with advanced HIV infection and/or long-term ART seen clinically. Our study shows antiretroviral compounds may effectively increase Aβ generation while possessing the capability to inhibit its clearance by preventing microglial phagocytosis. By affecting both amyloidogenic fronts (generation and clearance), antiretroviral treatment may substantially enhance Aβ aggregation and deposition, which itself is neurotoxic.

We find the most significant amyloidogenic effects when the antiretroviral compounds 3TC, Indinavir, and Abacavir are used in combination (Figure 1). Also, we observe that particular antiretrovirals, Indinavir and Abacavir, may have detrimental additive effects on Aβ microglial clearance (Figure 2). Accordingly, it is likely that certain 3 drug regimens may present an even greater risk of neurological complications.

Though higher CNS-penetrating regimens have been associated with neurocognitive improvement, recent research demonstrates ART might also impart neurotoxic effects; adversely affecting cognition. Indeed in a recent clinical study discontinuation of ART in experienced subjects improved neurocognition and those results were not attributed to practice effects. Furthermore, subjects re-initiated on ART did not experience cognitive gains. Therefore, ART neurotoxicity might explain the unexpected results of this clinical study gains [8]. Past reports have indicated the deleterious effects of ART on the brain were at least in part caused by damage to peripheral neural tissues (for review see [9]). NRTIs have been shown to induce toxicity in peripheral tissues by altering mitochondrial function, and PIs were shown to damage proteosome function [12,18]. Also, subjects on didanosine and stavudine regimens had decreased N-acetylaspartate (NAA) concentrations in frontal white matter, a sign of neurotoxicity which positively correlated with treatment duration [19].

Recently, studies have addressed the influence of five NRTIs (2' 3'-dideoxyinosine, zidovudine, emtricitabine, and tenofovir), one NNRTI (efavirenz), and two PIs (ritonavir, atazanavir sulfate) on neuronal integrity and function. All of the antiretroviral medications tested except for 2' 3'-dideoxyinosine reduced mitochondrial membrane potential. Furthermore, several antiretroviral medications destabilized neuronal intracellular calcium homeostasis, showing a reduced acute response to glutamate [20]. The ability of certain antiretroviral medications and combinations thereof to dysregulate neuronal calcium homeostasis and affect the mitochondrial membrane potential both promote the deposition of Aβ plaques and increased amyloidogenic processing of APP (for review see [21]). Furthermore, neurons treated with antiretroviral medications exhibited dendritic beading and pruning correlated over a range of doses, which has been linked to cognitive dysfunction [22].

In our experiments, the median toxic doses for several ARVs were well within the therapeutic concentration range in plasma of HIV-infected patients, and a few showed some signs of damage in the range of CSF concentrations. These initial observations highlight potential adverse effects of high concentrations of antiretroviral medications in the CNS and indicate that there may be some negative tradeoffs to traditionally delivering "therapeutic concentrations" of these compounds to the CNS.

As previous pharmacokinetic studies have confirmed the moderate to high oral bioavailabilities and low to moderate plasma protein binding properties of 3TC, Indinavir, and Abacavir, it is feasible that all of these antiretroviral compounds can reach systemic peak concentrations >100 uM following normal dosing regimens. Needless to say, only a fraction of these concentrations needs to be distributed in the brain to mediate the amyloidogenic effects we observed in our *in vitro *studies, which employed 10uM concentrations. Furthermore, ART effects *in vivo *are likely to occur over long-term exposures. Chronic, low dose, *in vivo *effects of any reagent are often very appropriately modeled *in vitro*, by proportionately higher doses of that same reagent, over more acute time frames [23]. For these reasons we used 10uM ART dose for these experiments.

This brings us to a potential dilemma in ART concerning an important parameter, blood brain barrier (BBB) permeability. On one hand 3TC, Abacavir, and Indinavir have been reported to be moderately BBB permeable and consequently may be free to promote amyloidosis. On the other hand, these antiretroviral compounds may also be more capable of reducing HIV load in the brain, which may be essential to avoid general HIV encephalopathy. Furthermore, Liu and colleagues [24] demonstrated that the HIV-1 Tat protein competitively inhibits the LRP receptor, resulting in an inhibition of Aβ clearance. Therefore, by minimizing HIV replication and associated Tat protein expression in the brain, BBB permeable antiretroviral compounds may also prevent amyloidosis. In light of this complex situation, switching from an ART regimen that is associated with both amyloidosis and the potentially related lipodystrophy to one without these adverse effects may be the best course of clinical action to reduce the risk of neurological complications. On the other hand, consistent association of neurocognitive impairment with nadir CD4 across pre-and post-ART eras suggests that earlier treatment to prevent severe immunosuppression may also help prevent HAND. Clinical trials targeting HAND prevention should specifically examine timing of ART initiation [2].

Future studies are also warranted to investigate the dose-response effects of newer BBB permeable antiretroviral drugs including fusion inhibitors, alone and in combination, on microglial CNS Aβ levels in an *in vivo *murine model. Moreover, future studies will be required to determine the effect of antiretrovirals on APP proteolysis and microglial Aβ proteases including neprilysin, insulin-degrading enzyme, and endothelin-converting enzymes 1 and 2, as ART modulated proteolytic activity could affect the development of HAND and amyloidosis. This study was also limited to the effect of ART of microglia and neurons separately without other mitigating factors. In the future this work will need to be replicated *in vivo *in mouse models to not only account for astrocytes, but the entire brain milieu. Nevertheless, it is clear from the most recent update by the World Health Organization in 2010 that the cost of rapid ART scale-up is significant in terms of side-effects [16]. There is a need from patients and health-care providers to phase in less toxic ART regiments while maintaining simplified fixed-dose combinations.

## Competing interests

The authors declare that they have no competing interests.

## Authors' contributions

BG and RS drafted the manuscript. JE and DO carried out the immunoassays. JJ, LL, and LL performed statistical analysis. FF conceived of the study. JT participated in its design and coordination. All authors read and approved the final manuscript.
